# Low-molecular-weight organic acids correlate with cultivar variation in ciprofloxacin accumulation in *Brassica parachinensis* L.

**DOI:** 10.1038/s41598-017-10701-7

**Published:** 2017-08-31

**Authors:** Hai-Ming Zhao, Lei Xiang, Xiao-Lian Wu, Yuan-Neng Jiang, Hui Li, Yan-Wen Li, Quan-Ying Cai, Ce-Hui Mo, Jie-Sheng Liu, Ming-Hung Wong

**Affiliations:** 1 Guangdong Provincial Research Center for Environment Pollution Control and Remediation Materials, School of Environment, Jinan University, Guangzhou, 510632 China; 2 College of Life Science and Technology, Jinan University, Guangzhou, 510632 China; 3 Department of Science and Environmental Studies, The Education University of Hong Kong, Hong Kong, China

## Abstract

To understand the mechanism controlling cultivar differences in the accumulation of ciprofloxacin (CIP) in Chinese flowering cabbage (*Brassica parachinensis* L.), low-molecular-weight organic acids (LMWOAs) secreted from the roots of high- and low-CIP cultivars (*Sijiu* and *Cutai*, respectively) and their effects on the bioavailability of CIP in soil were investigated. Significant differences in the content of LMWOAs (especially maleic acid) between the two cultivars played a key role in the variation in CIP accumulation. Based on the Freundlich sorption coefficient (*K*_*f*_) and distribution coefficient (*K*_*d*_), the presence of LMWOAs reduced the CIP sorption onto soil particles, and higher concentrations of LMWOAs led to less CIP sorption onto soil. On the other hand, LMWOAs enhanced CIP desorption by lowering the solution pH, which changed the surface charge of soil particles and the degree of CIP ionization. LMWOAs promoted CIP desorption from soil by breaking cation bridges and dissolving metal cations, particularly Cu^2+^. These results implied that the LMWOAs (mainly maleic acid) secreted from *Sijiu* inhibited CIP sorption onto soil and improved CIP desorption from soil to a greater extent than those secreted from *Cutai*, resulting in higher bioavailability of CIP and more uptake and accumulation of CIP in the former.

## Introduction

Antibiotics are used globally to treat disease and protect the health of humans and animals. The widespread use of antibiotics has raised concerns about their residues in the environment and food supplies. In recent years, concerns over the potential health risk and ecological effects of antibiotics in agricultural soils and crops have increased. Among the various antibiotics in use, fluoroquinolones are extremely effective antibacterial agents, and are used extensively in stockbreeding, leading to heavy soil pollution when fertilized with livestock manure^1, 2^. Ciprofloxacin (CIP) is one of the most frequently detected fluoroquinolone antibiotics in various environmental samples^3, 4^. The residues of CIP have been widely detected in agricultural soils at levels in excess of 100 μg kg^−1^, which is the ecotoxic effect trigger value set by the Steering Committee of the Veterinary International Committee on Harmonization^1, 5, 6^. Antibiotics in soil can be taken up by vegetables, potentially having adverse effects on human health via the food chain^1, 7, 8^. Therefore, how to reduce the accumulation of antibiotics in vegetables is an important issue for food safety and human health and requires further study.

The screening and breeding of pollutant-safe cultivars (PSCs) that accumulate low levels of pollutants in their edible parts has been proposed as a practical solution to guarantee food safety^9–12^. In recent years, numerous studies have been conducted to screen for PSCs, typically targeting heavy metals in many staple crops, including rice^10, 13^, wheat^14^, soybean^10^, etc. as well as in several leafy vegetables including Chinese cabbage^9^, Chinese flowering cabbage^15^, water spinach^11^, etc. Furthermore, the PSCs screened for heavy metals have been successfully used in the field^16^. However, few studies have reported the PSCs of organic contaminants, especially antibiotics. In our previous study, some crop cultivars that accumulated low levels of antibiotics were identified, including water spinach (*Ipomoea aquatic*) and Chinese flowering cabbage (*Brassica parachinensis* L.)^17, 18^. Nevertheless, the mechanism controlling the limited accumulation of antibiotics in PSCs remains poorly understood.

The uptake and accumulation of organic contaminants by crops are significantly affected by root exudates in soil^19^. Root exudates can enhance the mobility of organic contaminants in the rhizosphere and further uptake by plants^20–22^. Low-molecular-weight organic acids (LMWOAs), as essential components of root exudates, can disrupt the sequestration of contaminants by soil, thereby enhancing the bioavailability of organic contaminants in soil^19, 21^. The most common LMWOAs identified in the rhizosphere include oxalic, benzoic, succinic, lactic, malic, and citric acids^21, 23^. Nevertheless, the content and composition of LMWOAs released from roots are highly variable and dependent on plant species, cultivars, and the physiochemical environment^24^. The content and composition of LMWOAs may play a role in determining the bioavailability and uptake of pollutants by crop cultivars. Wang *et al*.^25^ reported that high-Hg cultivars of rice tended to secrete higher levels of LMWOAs in the rhizosphere compared with low-Hg cultivars, which enhances the bioavailability of Hg for root uptake. Xin *et al*.^26^ found that a low-Cd cultivar of hot pepper excreted significantly less tartaric acid and more oxalic and acetic acids than a high-Cd cultivar, suggesting that differences in Cd uptake and accumulation between the two cultivars might be modified by the changes in LMWOAs. However, the roles of LMWOAs in antibiotic uptake and accumulation in crops are still unclear, and the mechanisms by which LMWOAs account for crop cultivar variation in antibiotic accumulation have never been reported.

Sorption-desorption behavior is considered to be one of the most important processes influencing the bioavailability of contaminants in soil^26, 27^. Many studies have demonstrated that the LMWOAs of plants have an effect on the sorption-desorption behavior of organic contaminants^19–21, 27, 28^. For example, Luo *et al*.^27^ reported that LMWOAs could enhance the desorption of dichlorodiphenyltrichloroethane (DDT) from soil through partial dissolution of the soil structure, including the desorption of organic carbon from soils, and the formation of dissolved complexes with inorganic metal ions. Gao *et al*.^29^ found that the addition of LMWOAs enhanced the bioavailability of phenanthrene, but was dependent on the concentrations and types of LMWOAs in the soil, the aging time, and other factors. However, few studies have examined the effects of LMWOAs secreted from different crop cultivars on the sorption-desorption behavior of antibiotics in soil. The mechanisms involved in the effects of LMWOAs on antibiotic bioavailability may be partly responsible for the variation in antibiotic accumulation among crop cultivars.

Chinese flowering cabbage (*B*. *parachinensis* L.) is one of the most important leaf vegetables in southern China and is exported to many countries and regions around the world. Our previous study showed that high levels of fluoroquinolone antibiotics (mainly CIP) can be detected in this vegetable, posing a potential risk to human health^30^. Therefore, it is extremely important to identify PSCs for Chinese flowering cabbage with regard to CIP and to determine the mechanism of its low CIP accumulation. Based on our previous result^18^, two typical low- (*Cutai*) and high-CIP (*Sijiu*) cultivars of Chinese flowering cabbage were selected to investigate the content and composition of LMWOAs secreted by their roots. The effects of LMWOAs on the sorption-desorption behavior of CIP in soil were studied using a batch equilibration method. We hypothesized that the LMWOAs released from roots and their effects on the bioavailability of CIP in soil differ between the low- and high-CIP cultivars, which is partly responsible for the cultivar variation in CIP accumulation.

## Results

### CIP accumulation in the two cultivars

In the hydroponic experiment, concentrations of CIP in both shoots and roots and CIP net uptake via roots in the two cultivars increased substantially with increasing CIP levels. Under the same level of CIP exposure, *Sijiu* had significantly higher (*p* < 0.05) CIP concentrations in both shoots and roots and significantly higher (*p* < 0.05) CIP net uptake via roots than *Cutai* (Table 1). Although there was a significant difference (*p* < 0.05) in the transfer factors (TFs) of CIP between the two cultivars when exposed to 5 mg L^−1^ CIP, they were very low (<0.02; Table 1).

**Table 1:** Ciprofloxacin (CIP) concentration, translocation factor, and net uptake via roots for two cultivars of Chinese flowering cabbage exposed to CIP in a hydroponic culture for 40 days. * and ** indicate significant differences at *p* < 0.05 and 0.01 levels between the two cultivars, respectively, (*n* = 3).

CIP treatment (mg L^−1^)	Cultivar	CIP concentration (mg kg^−1^, DW)		CIP translocation factor	CIP net uptake via roots (mg kg^−1^, DW)
		shoot	root		
1	*Sijiu*	0.21 ± 0.02^*^	14.62 ± 1.21^*^	0.014 ± 0.002	15.67 ± 1.27^*^
	*Cutai*	0.15 ± 0.02	12.53 ± 0.74	0.012 ± 0.002	12.56 ± 0.86
5	*Sijiu*	0.82 ± 0.03^*^	120.03 ± 10.82^**^	0.007 ± 0.001^*^	124.34 ± 12.58^**^
	*Cutai*	0.61 ± 0.08	56.54 ± 41.83	0.011 ± 0.003	60.71 ± 5.63

*Note:* The CIP net uptake via roots was calculated as the ratio of the total amount of CIP in the whole plant (including the shoot and root) to root dry weight.

### Components and contents of LMWOAs in root exudates of the two cultivars

At different growth stages, both the components of LMWOAs and their contents excreted by roots of the cultivars were different. Four LMWOAs (maleic, tartaric, acetic, and malic acid) excreted by roots of the two cultivars under different CIP exposures were detected at the seedling stage, while five LMWOAs (maleic, tartaric, acetic, oxalic, and formic acid) were found at the flowering stage (Table 2). In general, the total content of LMWOAs produced by both cultivars increased with increasing CIP exposure at the seedling stage, indicating that CIP stress stimulated the release of LMWOAs. However, at the flowering stage, the release of LMWOAs at high levels of CIP exposure was inhibited when compared with that at low levels of CIP exposure. The total amount of LMWOAs secreted by *Sijiu* was always higher than that secreted by *Cutai* (Table 2).

**Table 2:** Differences in low-molecular-weight organic acid (LMWOA) concentrations between the two cultivars in control and ciprofloxacin (CIP) exposures (mg L^−1^ deionized water). S, seedling stage; F, flowering stage; * and ** indicate significant differences at the *p* < 0.05 and 0.01 levels between the two cultivars, respectively, (n = 3). nd, = not detected.

LOMWAs	Growth period	Control (0)		1 mg L^−1^ CIP		5 mg L^−1^ CIP	
		*Sijiu*	*Cutai*	*Sijiu*	*Cutai*	*Sijiu*	*Cutai*
Formic acid	S	nd	nd	nd	nd	nd	nd
	F	0.37 ± 0.11^**^	0.17 ± 0.01	0.15 ± 0.01	0.16 ± 0.03	0.25 ± 0.01	0.22 ± 0.03
Acetic acid	S	3.02 ± 0.33^*^	4.20 ± 0.67	2.81 ± 0.55	2.69 ± 0.15	3.68 ± 0.45^*^	3.09 ± 0.25
	F	3.79 ± 0.20^**^	0.64 ± 0.05	0.47 ± 0.04	0.41 ± 0.01	0.46 ± 0.07^*^	0.73 ± 0.12
Oxalic acid	S	nd	nd	nd	nd	0.34 ± 0.03	nd
	F	0.32 ± 0.05^**^	0.09 ± 0.02	0.41 ± 0.04^*^	0.26 ± 0.03	0.38 ± 0.03^**^	0.23 ± 0.02
Maleic acid	S	10.79 ± 2.70	8.71 ± 1.34	24.78 ± 1.22^*^	20.19 ± 2.00	52.27 ± 0.06^**^	22.89 ± 0.86
	F	14.73 ± 3.63^*^	7.50 ± 1.16	22.94 ± 0.88^**^	14.01 ± 0.92	14.98 ± 2.46^*^	11.97 ± 0.69
Tartaric acid	S	3.96 ± 1.56	3.04 ± 1.11	6.62 ± 1.27^*^	3.88 ± 0.55	12.33 ± 0.87^**^	4.24 ± 0.24
	F	2.74 ± 0.72	2.82 ± 0.53	8.21 ± 1.65^*^	14.09 ± 1.87	9.46 ± 1.02	9.84 ± 1.09
Malic acid	S	0.82 ± 0.03	0.82 ± 0.13	0.77 ± 0.04	0.72 ± 0.10	0.61 ± 0.06^*^	0.74 ± 0.02
	F	nd	nd	nd	nd	nd	nd
Total	S	18.59	16.77	37.98	27.48	69.23	30.96
	F	21.95	11.22	32.18	28.93	25.53	22.99

Maleic, acetic, and tartaric acids were the dominant LMWOAs detected in two growth stages for both cultivars. At all CIP levels and growth stages, maleic acid accounted for 50–70% of the total content of LMWOAs (Table 2). The maleic acid content varied with the same tendency as the total LMWOAs with increasing CIP levels, and the amount secreted by *Sijiu* was always significantly higher (*p* < 0.05) than the amount secreted by *Cutai* when exposed to CIP (Table 2). Although acetic and tartaric acids were detected at all CIP levels and growth stages, no significant variation was found between the two cultivars. The results indicated that a significant difference in the LMWOA content (mainly maleic acid) between the two cultivars might play a key role in their variation in CIP accumulation. Thus, maleic acid was selected as a representative of the LMWOAs to evaluate the effects of LMWOAs on the sorption-desorption behavior of CIP on soil. The artificial root exudates (AREs) were used as a positive control to simulate LMWOAs secreted from roots of Chinese flowering cabbage.

### Sorption kinetics of CIP onto soil particles

A series of contact time experiments for CIP (40 mg L^−1^) at temperatures of 25 ± 1 °C were conducted. The sorption kinetics obtained for the soil showed that the contact time necessary for CIP to reach equilibrium was 480 min (8 h) (Fig. S1a). The rate constant of sorption was determined from the pseudo-second-order equation based on equilibrium sorption expressed as^31^:

1 $$t/{q}_{t}=1/k{q}_{e}^{2}+t/{q}_{e}$$

where *q*_e_ and *q*_t_ are the amounts of CIP adsorbed (mg kg^−1^) at equilibrium and at time *t* (h), respectively, and *k* is the rate constant of sorption [kg (mg·h)^−1^]. The value of *k* [0.0125 kg (mg·h)^−1^] was calculated from the linear plots of *t*/*q*_t_ *vs*. *t* (Fig. S1b). The correlation coefficient for the second-order kinetic model was greater than 0.999, indicating the applicability of this kinetic equation and the second-order nature of the sorption process of CIP on soil.

### Effect of LMWOAs on the sorption and desorption of CIP

**Figure 1:**
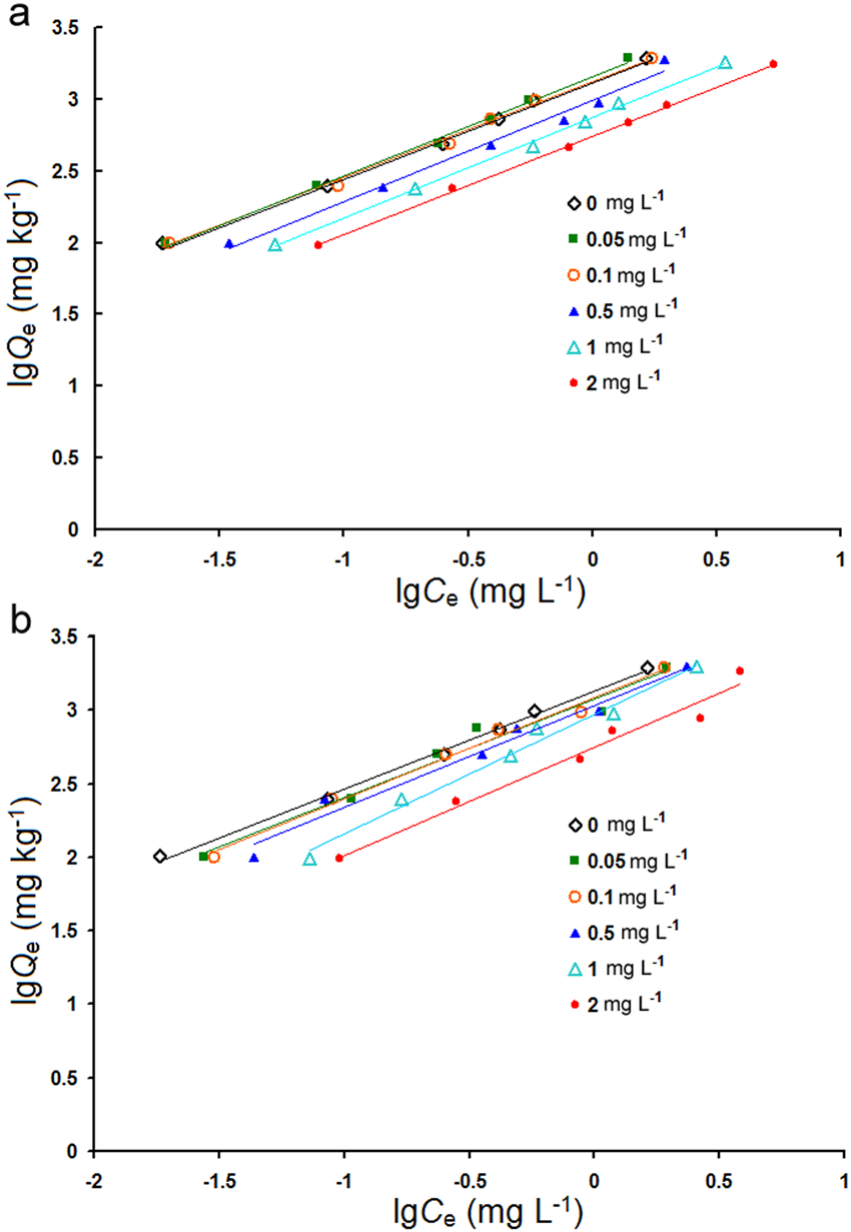
Freundlich isotherms fitted to describe CIP sorption onto soil particles in the presence of different concentrations of maleic acid (**a**) and artificial root exudates (AREs) (**b**).

**Figure 2:**
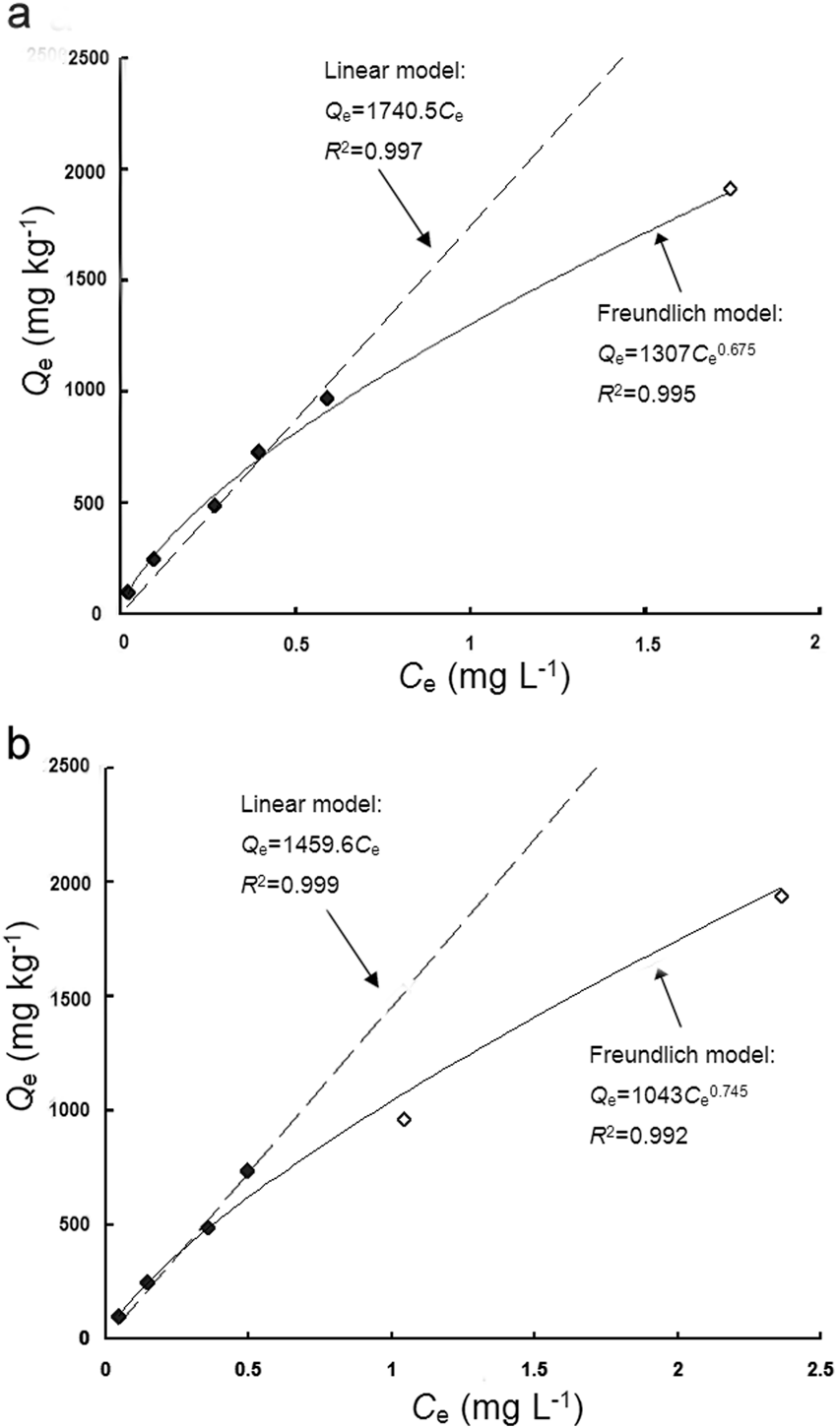
Representative linear sorption isotherms for calculating the *K*_*d*_ values for CIP at different concentrations of maleic acid (**a**) and artificial root exudates (AREs) (**b**). Dark symbols represent the low concentration used for the calculation of *K*_d_ values.

The CIP sorption isotherms (Fig. 1) were well described by the linearized form of the Freundlich equation (*R* ^2^ > 0.97). The calculated values of the Freundlich constants *K*_*f*_ and 1/*n* for CIP are given in Table 3. The addition of LMWOAs decreased *K*_*f*_ values from 1430 to 549 for maleic acid and from 1192 to 553 for AREs (Table 3). Over the entire concentration range, all Freundlich isotherms were observed to be nonlinear with 1/*n* coefficients less than unity. Thus, the distribution coefficient (*K*_*d*_) was calculated, using the linear part of the isotherm at low CIP concentrations, where 1/*n* was unity. Representative plots show the use of this approach in Fig. 2, where the *K*_*d*_ values represent different concentrations of LMWOAs (Table 3). The increased maleic acid or ARE concentrations tended to decrease the *K*_*d*_ values of CIP sorption at the same CIP concentration (Table 3), which showed that the presence of LMWOAs in a soil-water system impeded the distribution of CIP onto the soil solids. These findings indicate that the addition of LMWOA to soil decreased the sorption capacity of CIP, and that higher LMWOA concentrations generally led to marked decreases in CIP sorption by soil. It was noted that the *K*_*d*_ values of AREs were higher than those of maleic acid at various concentrations (Table 3), indicating greater effects of AREs on CIP sorption than maleic acid. In addition, the changes in free energy (Δ*G*) for CIP sorption on organic matter were calculated (Table 3). The Δ*G* changes ranged from −25.56 to −23.13 KJ mol^−1^ and from −25.05 to −23.15 KJ mol^−1^ at various concentrations of maleic acid and AREs, respectively. The negative values of Δ*G* in all cases indicated the spontaneous nature of CIP sorption on the soil.

**Table 3:** Values of thermodynamic parameters of the Freundlich models and linear models associated with ciprofloxacin (CIP) sorption onto soils at different concentrations of low-molecular-weight organic acids (LMWOAs). AREs, artificial root exudates.

LMWOAs	Concentration (g L^−1^)	Freundlich			Linear		*K*_*OM*_	*ΔG* (KJ mol^−1^)
		*K*_*f*_	1/*n*	*R* ^2^	*K*_*d*_	*R* ^2^		
Maleic acid	0	1325	0.667	0.997	1736	0.964	27263	−25.32
	0.05	1430	0.689	0.997	1816	0.960	29424	−25.56
	0.1	1307	0.675	0.995	1740	0.967	26893	−25.28
	0.5	971	0.704	0.987	933	0.933	19979	−24.55
	1.0	751	0.703	0.998	753	0.969	15453	−23.91
	2.0	549	0.684	0.999	478	0.946	11296	−23.13
AREs	0.05	1192	0.669	0.972	2133	0.991	24526	−25.05
	0.1	1179	0.692	0.991	1828	0.958	24259	−25.03
	0.5	1043	0.745	0.992	1459	0.984	21460	−24.72
	1.0	921	0.817	0.985	1172	0.971	18951	−24.42
	2.0	553	0.739	0.973	580	0.951	11379	−23.15

**Figure 3:**
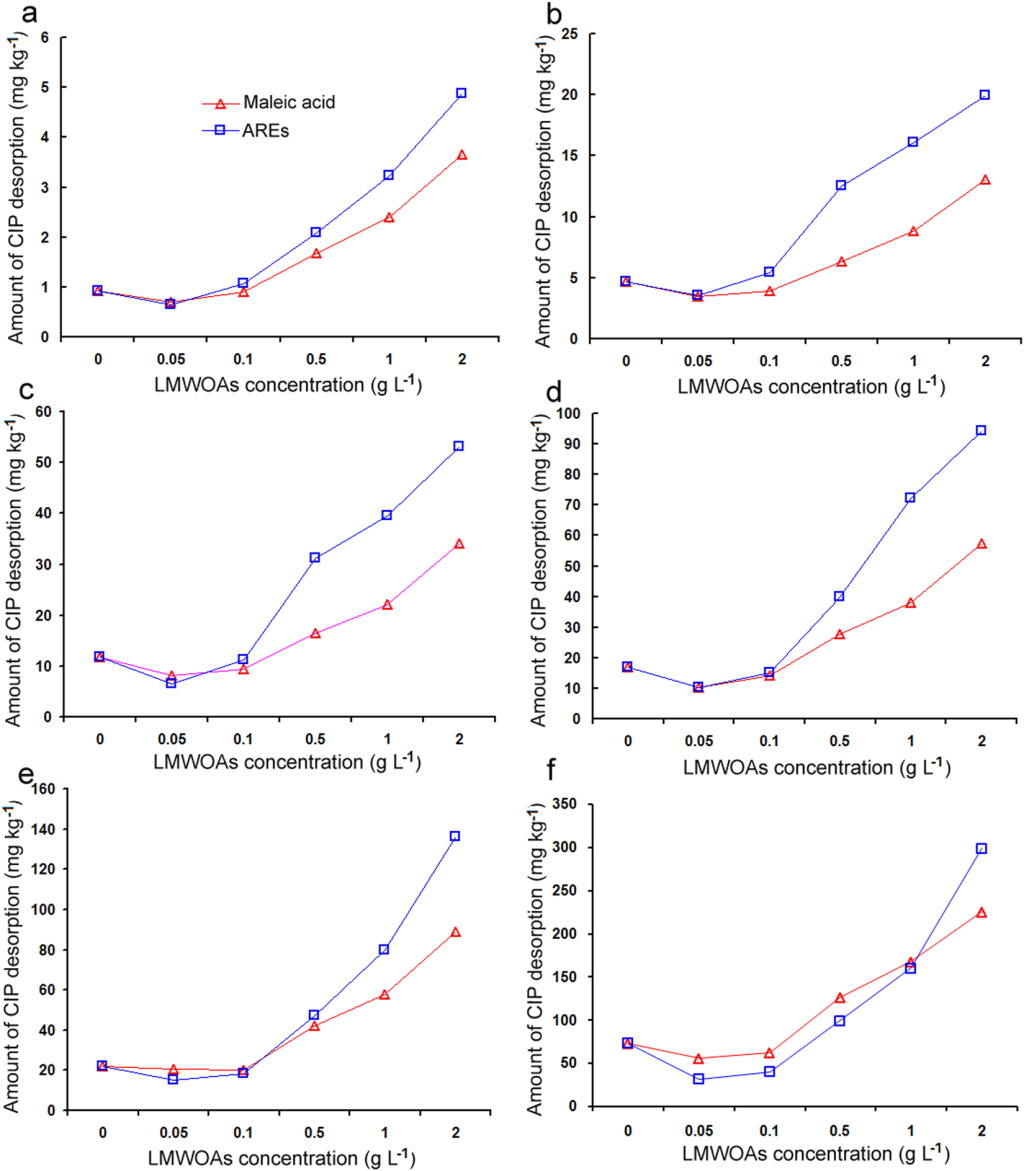
Effect of low-molecular-weight organic acids (LMWOAs) on CIP desorption from soil particles at initial loading of 99.1 (**a**) 245.7 (**b**) 487.4 (**c**) 728.8 (**d**) 970.9 (**e**) and 1918.0 (**f**) mg kg^−1^.

In the equilibrium solution, the amounts of CIP sorption onto soil particles were correlated with the initial concentrations of CIP (*R* ^2^ = 1.000) (Fig. S2). When the CIP initial concentrations were 2, 5, 10, 15, 20, and 40 mg L^−1^, the corresponding amounts of CIP equilibrium sorption were 99.1, 245.7, 487.4, 729.0, 970.9, and 1,918.0 mg kg^−1^, respectively. In this case, the amount of CIP desorption as a function of the LMWOA concentration (0–2.0 g L^−1^) is presented in Fig. 3. Generally, the addition of low levels of LMWOAs (0.05–0.1 g L^−1^) did not change the amount of CIP desorbed from soil particles, but the addition of high levels (0.5–2.0 g L^−1^) markedly increased the amount of CIP desorption. For example, at the initial loading of 99.1 mg kg^−1^ of CIP in the soil (Fig. 3a), the addition of 0.05 g L^−1^ maleic acid or AREs only slightly decreased the amount of CIP desorption (by 5.04% or 5.36%, respectively) compared to that in the control (0 g L^−1^), while the addition of 2.0 g L^−1^ maleic acid or AREs substantially increased the amount of CIP desorption by 297.83% or 429.35%. Thus, it could be concluded that the presence of LMWOAs promoted CIP desorption from soil particles, and that higher LMWOA concentrations facilitated greater CIP desorption. Additionally, more CIP desorption occurred at high concentrations (0.5–2.0 g L^−1^) of AREs than at high concentrations of maleic acid, which might be attributable to the more complicated composition of AREs. These results indicate that the effects of LMWOAs on CIP desorption may be predominantly due to maleic acid.

### Effect of pH on CIP desorption

It was found that the zeta potential of soil solution was substantially affected by pH (Fig. S3). The soil surface had a positive charge at pH 3.03, but it had a negative charge at pH 3.92, with little change as pH increased to 5.40. The isoelectric point of the soil solution was identified at pH 3.24. The addition of LMWOAs remarkably decreased the pH of soil solution with different CIP initial loadings (Table S1). The addition of maleic acid led to a greater decrease in pH than the addition of AREs, with the decrease being larger as the amount of maleic acid added became larger. When the concentrations of LMWOAs added were lower than 0.1 g L^−1^, the pH values of soil solutions were higher than the isoelectric point of the soil solution (Table S1). Thus, at 3.24 < pH < 6.10 (p*K*_a1_ of CIP), the soil surface was negatively charged, while CIP existed in the form of CIP^+^ cations (Fig. S4). The CIP^+^ cations were adsorbed to the negatively charged soil surface. When the LMWOA concentrations were higher than 0.5 g L^−1^ (maleic acid) or 1.0 g L^−1^ (AREs), the pH values of soil solutions were lower than 3.24 (Table S1), leading to the desorption of CIP^+^ from positively charged soil surfaces due to the natural repulsion of the same electric charge (Fig. 3). In theory, the effect of maleic acid addition on CIP desorption from soil should have been greater than that of AREs at a soil solution pH < 3.24, but the opposite results were observed (Fig. 3), implying that there were other factors leading to CIP desorption in addition to pH. Remarkable desorption of CIP was observed at pH > 3.24, when the concentration of AREs added was 0.5 g L^−1^ (Fig. 3).

### The release of metal ions in soil in the presence of LMWOAs

Five metal ions (Cu^2+^, Zn^2+^, Cd^2+^, Fe^3+^, and Mg^2+^) were determined in the equilibrium soil solutions in the presence of LMWOAs (Table S2). The release of metal ions, especially Cu^2+^, Zn^2+^, and Fe^3+^, increased substantially with increasing concentrations of LMWOAs in the equilibrium soil solution with different CIP loadings. A correlation analysis revealed that there were significantly positive correlations (*p* < 0.05) between the amount of CIP desorption and the amount of metal ions released, especially Cu^2+^, Zn^2+^, and Fe^3+^ (Table 4). The results from a multivariate regression analysis showed that Cu^2+^ was the main factor influencing CIP desorption.

**Table 4:** Best fitting equations of ciprofloxacin (CIP) desorption and metal ions released in the equilibrium soil solution with different CIP loadings. *x*, the amount of CIP desorption; *y*, the amount of metal ions released; *Q*, CIP desorption index; * and ** indicate significant differences at the *p* < 0.05 and 0.01 levels, respectively.

CIP loading (mg kg^−1^)	Cu	Zn	Cd	Fe	Mg	Multivariate regression
99.1	*y* = 3E-02*x* + 0.017^**^	*y* = 1E-02*x* − 0.006^*^	*y* =−7E-05*x* + 0.043	*y* = 2E -01*x*−0.173^*^	*y* =−3E-02*x* + 0.629	*Q* = 38Cu + 2.05Zn − 1.82Fe−0.87 *R* ^2^ = 0.954
970.9	*y* = 9E-05*x* − 0.005^**^	*y* = 3E-05*x* + 0.009	*y* =−5E - 07*x* + 0.040	*y* = 5E-05*x* − 0.026^*^	*y* =−3E-05*x* + 0.339	*Q* = 7614Cu + 4946Fe − 209 *R* ^2^ = 0.953
1918.0	*y* = 4E-05*x*−0.009^**^	*y* = 9E-05*x*−0.098	*y* = 5E - 07*x* + 0.042	*y* = 3E-04*x* − 0.431	*y* = 5E-05*x* + 0.317	*Q* = 26336Cu + 289 *R* ^2^ = 0.979

## Discussion

The production of vegetable crops that are safe for human consumption in slightly and moderately contaminated agricultural soils is an issue of increasing concern. Recently, the PSC strategy has attracted widespread attention^9–12^. Nevertheless, the identification and studies of PSCs with regard to organic contaminants are limited. In our previous pot experiment, a low-CIP cultivar (*Cutai*) of Chinese flowering cabbage was identified and its CIP concentration in shoot was found to be lower by 9.6-fold than that of a high-CIP cultivar (*Sijiu*) when grown in CIP-contaminated soils^18^. However, the difference in the shoot CIP concentration between the two cultivars was only 1.3- to 1.4-fold in our hydroponic experiment (Table 1). This might have been due to the higher bioavailability of CIP in hydroponic conditions than in soil, which made it easier for *Cutai* to take up and accumulate CIP. That is, CIP is completely available for root uptake of the two cultivars in hydroponic condition. However, the availability of CIP in soils is controlled by adsorption and desorption characteristics of soils, associating with many interacting factors (e.g., soil properties, pH value, moisture content, etc.)^32^. Thus, their pronounced difference in the shoot CIP concentration may be attributed to changed bioavailability of CIP in soils caused by the different rhizosphere effect between the two cultivars. Zornoza *et al*.^33^ reported that Hg bioavailability was a major factor limiting Hg uptake and accumulation by roots of white lupin in different substrates, leading to more Hg accumulation in hydroponically grown plants than in soil-grown plants. In this study, the very low TFs (<0.02) in both cultivars failed to clarify the mechanism causing the cultivar variation in CIP accumulation. This was different from the mechanisms of phthalate ester (PAE) or heavy metal accumulation in some crop cultivars, where TFs (>0.1) have been shown to play a key role in the variation in pollutant accumulation among cultivars^9, 11, 14, 15^. Moreover, significant differences (*p* < 0.05) in root CIP uptake between *Sijiu* and *Cutai* were observed (Table 1), demonstrating that root uptake played an important role in the variation in CIP accumulation between the two cultivars. The bioavailability of CIP in the rhizosphere for root uptake was one of the major reasons for the difference in CIP accumulation between the two cultivars.

The bioavailability of organic contaminants in the rhizosphere is an important factor limiting their uptake by plants^21, 29^. Root exudation plays an important role in influencing the bioavailability of organic contaminants in rhizospheric soil by changing soil properties, microbial activity, and soil enzyme activity^20, 29^. LMWOAs originating primarily from root exudation occur widely in rhizospheric soil. They promote the dissolution of soil phases and facilitate the uptake of dissolved compounds by plants through chelation and H^+^-promoted reactions because they possess one or more carboxyl and hydroxyl groups^28^. Accordingly, LMWOAs can exert an important influence on the bioavailability of organic contaminants in the rhizosphere of plants^19–21^. In the present study, there were clear differences in the composition and concentrations of LMWOAs between the two cultivars (Table 2). The higher total LMWOA content in *Sijiu* was associated with more dissolved CIP, leading to more accumulation of CIP in *Sijiu* than in *Cutai*. This result was consistent with the report of Liu *et al*.^34^, who found that LMWOA concentrations in the rice cultivar *Shanyou63* (a high Cd accumulator) were higher than those in *Wuyunjing7* (a low Cd accumulator).

The main LMWOAs in both cultivars of Chinese flowering cabbage were maleic, acetic, and tartaric acids. Their concentrations varied with CIP stress and growth stages, indicating that they were very important for plant growth in response to environmental stresses^35^. In all of the LMWOAs examined, maleic acid, which accounted for over 50% of the total LMWOAs, was always higher in *Sijiu* than in *Cutai* (Table 2), indicating that maleic acid was an important factor influencing CIP accumulation. A similar result was found in the rhizosphere of water spinach, where maleic acid was the dominant (52.2–56.6%) LMWOA and controlled the accumulation of many heavy metals (e.g., Cd, Cr, Cu, Pb, and Zn) among different cultivars^35^. In contrast, Xin *et al*.^24^ reported that succinic acid accounted for the largest proportion (43.7–73.6%) of LMWOAs excreted from hot pepper roots under Cd stress, and its effects on the uptake, translocation, and accumulation of Cd in hot pepper depended upon the cultivar and Cd supply concentrations. The different results reflected differences in plant species or cultivars that had specific LMWOA exudation mechanisms, accounting for the overall variation in pollutant accumulation. The differences in CIP accumulation between the Chinese flowering cabbage cultivars may be partly attributable to the concentration of maleic acid in root exudates. The detailed effects of maleic acid on the bioavailability of CIP in soil need to be investigated to identify the mechanism that controls the variation in CIP accumulation among different cultivars.

The rate of sorption and desorption of an organic compound to and from soil particles determines its bioavailability in natural environments^29^. Many studies have indicated that LMWOAs could alter the bioavailability of organic compounds in soil through batch equilibrium assays^19, 20, 27–29^. Zhang *et al*.^36^ reported that the LMWOAs, including citric acid, malic acid, and salicylic acid, inhibited the norfloxacin sorption process by changing the soil pH, surface charge of soil particles, and competitive sorption of co-existing cations. Accordingly, sorption-desorption experiments were performed in our study to assess the effect of LMWOAs on the bioavailability of CIP in rhizosphere soil. It was found that LMWOAs, including maleic acid and AREs, enhanced the bioavailability of CIP by inhibiting sorption and promoting desorption of CIP in soil (Table 3 and Fig. 3). Effective sorption for selected LMWOAs was modeled in two different manners, with Freundlich isotherm parameters (*K*_*f*_ and 1/*n*) and linear distribution coefficients (*K*_*d*_) being obtained. The CIP sorption onto soil in the presence of LMWOAs produced L-type isotherms (1/*n* < 1), suggesting a strong interaction between soil and CIP molecules and a decreasing sorption tendency with increasing equilibrium concentrations^37^. To avoid underestimating the importance of low-CIP loading data, *K*_*d*_ values were also calculated by a linear fit^38^. *K*_*d*_ values followed the same trend as that observed for the *K*_*f*_ values (Table 3). Based on the observed *K*_*f*_ and *K*_*d*_ values, these results indicated that the presence of LMWOAs in soil reduced the amount of CIP sorption, and that higher LMWOA concentrations generally led to marked decreases in CIP sorption by soil. Furthermore, it was found that the Δ*G* values were <40 KJ mol^−1^, demonstrating that CIP was physically adsorbed on the soil^39^. The Δ*G* values increased gradually with increasing LMWOA concentration (Table 3), indicating that the spontaneity of CIP sorption on soil was inhibited. Hence, the increase in Δ*G* after the addition of LMWOAs further confirmed the role of LMWOAs in restraining the sorption of CIP onto soil. On the other hand, the addition of LMWOAs significantly increased CIP desorption from soil, and the higher concentration of LMWOAs led to more desorption of CIP in soil solution (Fig. 3). Therefore, the high-CIP cultivar *Sijiu* secreted more LMWOAs than the low-CIP cultivar *Cutai*, with the result that the CIP became more bioavailable due to it being desorbed from rhizosphere soil and becoming available for root uptake. In addition, AREs promoted CIP desorption to a greater degree than maleic acid, especially when high concentrations were added (Fig. 3), which might result from more complex components in AREs. These results were consistent with the reports that LMWOAs remarkably influenced the desorption of polycyclic aromatic hydrocarbons (PAHs), depending on the type and concentration of LMWOA, with citric acid enhancing the desorption of PAHs more efficiently than other LMWOAs^21, 28, 29^. In the present study, we found that maleic acid was the main LMWOA affecting the sorption-desorption behavior of CIP.

CIP desorption was highly dependent on solution pH, which affected the surface charge of soil and the degree of ionization of CIP^40, 41^. As shown in Fig. S4, CIP exists as a cation, neutral, and/or anion species under environmentally relevant pH conditions. In the present study, the pH of the soil solutions with LMWOA addition ranged from 5.38 to 2.06 for maleic acid and from 5.41 to 2.45 for AREs (Table S1), and the isoelectric point of the soil solution was 3.24 (Fig. S3). Therefore, as indicated by its p*K*_a_ values and its ionization (Fig. S4), CIP existed in the form of a cation (pH < p*K*_a1_) in all equilibrium solutions with LMWOA addition. With the decrease of pH to 3.24 with maleic acid addition, the opposite charges led to an attraction between CIP^+^ and negatively charged soil surfaces (pH > 3.24), and no CIP desorption occurred. At pH < 3.24, desorption occurred due to electrostatic repulsion between CIP^+^ and the positive surface of soil particles, which was confirmed by the results of the CIP-desorption experiment (Fig. 3). This explained how maleic acid could influence the CIP-desorption process by altering the pH of the soil solution. A similar trend of CIP desorption was observed with ARE addition (Fig. 3). Remarkable desorption of CIP was observed at pH > 3.24 when the concentration of AREs added was 0.5 g L^−1^ (Fig. 3), suggesting that pH was not necessarily a key factor controlling CIP desorption from soil in the presence of LMWOAs.

It has been reported that the desorption of CIP is controlled by various factors, such as coexisting metal cations and dissolved organic matter^38–40^. Metal cations in soils form complexes with functional groups of organic molecules, leading to the formation of “bridges” between the soil solid surface and the organic compound^20, 42^. Tan *et al*.^39^ reported that metal cations (Cu^2+^, Pb^2+^, Cd^2+^, and Ca^2+^) enhance the CIP sorption on goethite surfaces by an ion bridging effect to form goethite-metal-CIP ternary surface complexes. However, it has been reported that LMWOA addition could enhance the release of organic compounds from these complexes by breaking the bridges and dissolving the metal cations into solution^19, 39^. In the present study, five metal ions (Cu^2+^, Zn^2+^, Cd^2+^, Fe^3+^, and Mg^2+^) were determined in soil solution in the presence of LMWOAs. There were significantly positive correlations (*p* < 0.05) between the amount of metal ions released, especially Cu^2+^, and the amount of CIP desorption. A multivariate regression analysis showed that Cu^2+^ was the main factor influencing CIP desorption following the addition of LMWOAs (Table 4). Compared to other metal ions, it was found that Cu^2+^ had a stronger binding affinity to CIP to form the goethite-Cu-CIP complex as a “cation bridge”^39^, which might be the same mechanism that operated in this study. In a soil/metal ion/CIP ternary system, the adsorption of CIP is increased when it coexists with Cu^2+^ through the formation of a soil-Cu^2+^-CIP ternary surface complex. The effect of Cu^2+^ on CIP binding is pH-dependent. The maximum adsorption of CIP with Cu occurred at approximately pH 6, and its adsorption capacity decreased with decreasing pH^39^. In the present study, the addition of LMWOAs decreased the pH of soil solutions (Table S1) and consequently enhanced CIP desorption from soil by breaking the “Cu bridge” and dissolving Cu^2+^ into solution. It is interesting to note that more CIP desorption was observed with ARE addition than with maleic acid addition when the concentration of LMWOAs added was greater than or equal to 0.5 g L^−1^ (Fig. 3). It is likely that the combination of various LMWOAs as AREs had a marked “additive” effect on the mobilization of CIP. This was consistent with a previous report describing that the capacity of citric, malic, and oxalic acids to desorb P was significantly greater in cocktails than a single LMWOA due to the interactive effects of the LMWOAs^43^. Further work is also required to obtain a better understanding of why some plant species or cultivars exude a complex mixture of LMWOAs at high release rates, while others release them predominantly in isolation. Thus, more in-depth studies of the role of LMWOAs in controlling the variation in CIP accumulation among crop cultivars are needed.

## Methods

### Plant cultures and collection of root exudates

According to previous reports, CIP concentration has been identified up to milligram levels in agricultural soils^1, 44^. Thus the hydroponic solution spiked at 1.0 mg L^−1^ was used in line with the actual concentration in field. Additionally, the solution spiked at 5.0 mg L^−1^ was set to evaluate the differential responses of the two cultivars to higher level of CIP stress. Uniform seedlings of *Sijiu* and *Cutai* bred in sand culture were selected and transplanted into aerated hydroponic solutions spiked with the two levels of CIP (1.0 and 5.0 mg L^−1^). The same treatment without CIP served as a control. Five replicates were used for each treatment. Plants were cultured under greenhouse conditions with a 12-h light period at 25–35 °C. Nutrient solutions with and without CIP treatment were replaced every two days, and the loss of water evaporation in solution was replenished by adding deionized water every day. On the 12^th^ day (seedling stage) and 40^th^ day (flowering stage) after transplanting, root exudates were collected 2 h after the beginning of the photoperiod according to Lu *et al*.^45^ with minor modifications (see supplementary information).

### CIP and LMWOA analyses

After freeze-drying, CIP in vegetable samples was extracted and determined by HPLC-MS/MS following the method used in our previous studies^6, 30^ (see supplementary information). Recoveries of spiked CIP in vegetables ranged from 86.9% to 90.1%. The limit of detection and limit of quantitation were 0.024 and 0.084 μg kg^−1^, respectively. LMWOAs in samples were determined by ion chromatography (IC) equipped with an Ion IonpacAS11-HC column and an IonpacAG11-HC guard column (50 × 4 mm). The mobile phase was KOH solution at a flow rate of 1.0 mL min^−1^. The column temperature was 30 °C, and the injection volume was 1.0 mL. The gradient elution process was 1.0 mM for 10 min, 45.0 mM for 25 min, and 1.0 mM for the final 5 min. LMWOAs were detected by comparing retention times and absorption spectra with standards of seven organic acids (malic, acetic, formic, maleic, tartaric, oxalic, and citric acids).

### Sorption-desorption experiments

A batch equilibration method was used to investigate the sorption and desorption behaviors of CIP in soil-water systems. The amount of CIP sorption on soil particles as a function of time was determined to evaluate the equilibration time. A 0.5-g soil sample (DW) was mixed into 25 mL of CIP solution (40 mg L^−1^) in a 50-mL Teflon centrifuge tube and shaken on a reciprocating shaker at 200 rpm at 25 ± 1 °C for 36 h. To maintain constant ionic strength and inhibit microbial growth in the soil-water system, we added 0.01 M CaCl_2_ and 0.01 M NaN_3_, respectively. After 5, 10, 20, 30, 60, 120, 240, 480, 960, 1,440, and 2,160 min, triplicate tubes were sampled and centrifuged at 6,000 rpm for 10 min. The supernatant was filtered through a 0.45-μm filter, and the CIP concentration was determined by ultra-pure liquid chromatography (UPLC). Our preliminary experiment showed that no CIP degradation and sorption to the tubes occurred during equilibration.

The effects of LMWOAs on the sorption of CIP to soil particles were examined by performing sorption experiments using maleic acid and AREs. A 0.5-g soil sample was mixed into 25 mL of CIP solution with different initial concentrations (2, 5, 10, 15, 20, and 40 mg L^−1^) that contained LMWOAs, CaCl_2_, and NaN_3_. The amount of LMWOAs (maleic acid or AREs) in CIP solutions was set at 0, 0.05, 0.1, 0.5, 1.0, and 2.0 g L^−1^, respectively. The solutions were shaken in the dark at 200 rpm and 25 ± 1 °C for 8 h, and then centrifuged at 6,000 rpm for 10 min. The supernatant was filtered through a 0.45-μm filter, and the CIP concentration was determined by UPLC. The amount of CIP sorption on soil was calculated from the differences between the initial and final concentrations in solution.

To measure CIP desorption in the presence of LMWOAs, 0.5 g of soil was mixed into 25 mL of CIP solutions with different initial concentrations (2–40 mg L^−1^, containing CaCl_2_ and NaN_3_) and shaken in the dark at 200 rpm under 25 ± 1 °C for 8 h to reach equilibrium. Then, all of the supernatants were removed and replaced by the same volume of LMWOA solutions (maleic acid or AREs, 0–2.0 g L^−1^), followed by shaking, centrifugation, and CIP analysis as described above. The amount of CIP remaining after desorption was calculated from the difference between the amount of equilibrium sorption and the CIP concentration in the supernatant. The pH and concentration of metal ions (Cu^2+^, Zn^2+^, Cd^2+^, Fe^3+^, and Mg^2+^) in the supernatant were determined using a pHS-3C pH meter (Leici, Shanghai, China) and atomic absorption spectrometry (AA7000; Shimadzu, Tokyo Japan), respectively. All tests and analyses were conducted in triplicate.

### Thermodynamic analysis

CIP sorption to the soil was described using the Freundlich equation. The nonlinear form of the Freundlich sorption model was expressed as:

2 $${Q}_{e}={K}_{f}{C}_{e}^{1/n}$$

where *Q*_*e*_ is the amount of CIP adsorbed (mg kg^−1^) onto soil under equilibration, *C*_*e*_ is the CIP equilibrium concentration (mg L^−1^) in the aqueous phase, and *K*_*f*_ and 1/*n* are the sorption coefficient and the Freundlich measure of nonlinearity, respectively. The linearized form of the Freundlich isotherm can be expressed as:

3 $$\mathrm{lg}\,{Q}_{e}=\,\mathrm{lg}\,{K}_{f}+1/n\,\mathrm{lg}\,{C}_{e}$$

The CIP sorption isotherms at different concentrations of LMWOAs were determined from linearized plots of lg*C*_*e*_ *vs*. lg*Q*_*e*_, as per eq. (2).

At low CIP loadings and with a lack of apparent sorption site saturation, the distribution coefficient (*K*_*d*_, L kg^−1^) was calculated using only the initial linear part of the isotherm plotted as *C*_*e*_ *vs*. *Q*_*e*_^46^. Note that when the Freundlich 1/*n* is unity, the Freundlich sorption coefficient (*K*_*f*_) is equal to the distribution coefficient (*K*_*d*_). For the linear portion of the isotherm, the model may be expressed as:

4 $${K}_{d}={Q}_{e}/{C}_{e}$$

The sorption constant (*K*_*OM*_) of organic matter was calculated using the organic matter content (*ω*_*OM*_) of the soil and the following equation^46^:

5 $${K}_{OM}=1000\,K{}_{f}/{\omega }_{OM}$$

Assuming that CIP sorption was mainly due to organic matter, the change in free energy for sorption was calculated using the value of *K*_*OM*_ and the following equation:

6 $${\rm{\Delta }}G=-RT\,\mathrm{ln}\,{K}_{OM}$$

where Δ*G* is the free energy change (cal mol^−1^), *R* is the gas constant [8.314 J (K·mol^−1^)], and *T* is the Kelvin temperature.

### Zeta potential measurement

The effect of pH on the ζ (zeta) potential of soil particles was evaluated using a Zetasizer Nano ZS (Malvern Instruments, Malvern, UK). A series of 0.01-g soil samples were transferred into beakers containing 50-mL aqueous solutions with different pHs and mixed homogeneously by stirring in an ultrasonic bath (SK2000LH; Kedao, China). The time *vs*. pH results indicated that the pH of the solution stabilized after 30 min. After being left to stand for 2 h to reach equilibrium, the pH and zeta potential of the solution were measured. The zeta potential of at least six soil samples for each pH solution was determined and the average was recorded. The temperature of the room was 25 ± 1 °C.

### Statistical analysis

The CIP uptake ability of plants can be estimated by the net uptake of CIP via roots, which was calculated as the ratio of the total amount of CIP in the whole plant (including the shoot and root) to root dry weight^26^. The translocation factor (TF) was calculated as the ratio of the CIP concentration of the shoot to that of the root. Results are presented as mean ± standard error (SE). A correlation analysis, multivariate regression analysis, and one-way analysis of variance (ANOVA) followed by Duncan’s multiple-range test were performed with Microsoft Excel 2003 and SPSS 17.0 (SPSS Inc., Chicago, IL, USA).

## Electronic supplementary material

Supplementary Information
