# Supplementary material for: Low-molecular-weight organic acids correlate with cultivar variation in ciprofloxacin accumulation in Brassica parachinensis L
Source: Sci Rep. 2017 Aug 31;7:10301. doi: 10.1038/s41598-017-10701-7 (PMC5579271; doi:10.1038/s41598-017-10701-7)
Supplement: Supplementary file 1 — Supplementary Information [file 41598_2017_10701_MOESM1_ESM.docx]

**Supplementary Information**

**Low-molecular-weight organic acids correlate with cultivar variation in ciprofloxacin accumulation in *Brassica parachinensis* L.**

Hai-Ming Zhao^1^, Lei Xiang^1^, Xiao-Lian Wu^1^, Yuan-Neng Jiang^1^, Hui Li^1^, Yan-Wen Li^1^, Quan-Ying Cai^1^, Ce-Hui Mo^1,*^, Jie-Sheng Liu^2^, Ming-Hung Wong^1,3^

^1^Guangdong Provincial Research Center for Environment Pollution Control and Remediation Materials, School of Environment, Jinan University, Guangzhou 510632, China

^2^College of Life Science and Technology, Jinan University, Guangzhou 510632, China

^3^Department of Science and Environmental Studies, The Education University of Hong Kong, Hong Kong, China

***Corresponding Author**

(Ce-Hui Mo) Phone: +86 20 85220564. Fax: +86 20 85226615. E-mail: tchmo@jnu.edu.cn

**Supplementary materials and methods**

**Chemicals.** CIP was obtained from Dr. Ehrenstorfer-Schäfers (purity >98%; Augsburg, Germany). The molecular structure and selected physicochemical properties of CIP are shown in Fig. 1. Methanol and acetonitrile (HPLC grade) were obtained from Sigma-Aldrich (St. Louis, MO, USA). All other reagents were of analytical grade and were obtained from Damao Chemical Reagent Co. (Tianjin, China).

According to the analysis of LWMOAs released from the roots of Chinese flowering cabbage, the artificial root exudates (AREs) used in the sorption-desorption experiments consisted of [maleic](javascript:void(0);) [acid](javascript:void(0);), tartaric acid, [acetic](javascript:void(0);) [acid](javascript:void(0);), oxalic acid, malic acid, and formic acid at a ratio of 1:0.5:0.25:0.125:0.125:0.125.

**Plant and soil materials.** Seeds of two Chinese flowering cabbage cultivars that accumulated high and low levels of CIP (i.e., *Sijiu* and *Cutai*, respectively), were obtained from Guangdong Academy of Agricultural Science, China. The soil collected from the surface layer (0–20 cm) of an agricultural field (Guangzhou, China) was air-dried and ground to pass through a 2-mm sieve for sorption-desorption experiments. The main physiochemical properties of the soil were 48.6 g kg^−1^ (dry weight, DW) organic matter, 4.19 g kg^−1^ total N, 1.03 g kg^−1^ total P, 18.0 g kg^−1^ total K, 7.67 cmol kg^−1^ cation exchange capacity, 33.5% sand, 18.5% silt, 48% clay, and pH 7.09.

**Collection of root exudates.** Roots were rinsed thoroughly five times with deionized water to remove nutrients and CIP on the surface. Then, the roots were immersed in deionized water (500 mL) in dark conditions. After 9-h incubation, root exudates were collected, evaporated to dryness at 40°C using a vacuum rotary evaporator, and then dissolved in 5 mL of deionized water. The samples were filtrated through a 0.45-μm membrane and analyzed by ion chromatography (IC, ICS-900; Dionex, Sunnyvale, CA, USA). After the collection of root exudates, the plants were harvested (at day 40) and divided into roots and shoots for determining the dry weights and concentrations of CIP.

**CIP analysis by HPLC-MS/MS.** Briefly, a vegetable sample (1.0 g, DW) was extracted ultrasonically three times using 15 mL of acetonitrile-hydrochloric acid (125:8, V/V), and then purified and concentrated using Oasis HLB extraction cartridges (3 mL/60 mg; Waters, Milford, MA, USA). The extracts were determined by a high-performance liquid chromatography–tandem mass spectrometry system (HPLC–MS/MS: Alliance 1100 (HPLC), AB4000QTRAP (MS), Agilent, Santa Clara, CA, USA) using acetonitrile:water (10/90, V/V, with 0.1% formic acid) as the mobile phase at a flow rate of 0.2 mL/min. The column used was an Agilent Eclipse Plus C18 (5 μm, 2.1 × 150 mm), with a column temperature of 20°C and an injection volume of 5 μL. The instrument was operated in positive ion electrospray ionization (ESI) mode for multiple reaction monitoring (MRM). The desolvation temperature was adjusted to 600°C, ion source voltage was set at 5.5 kV, curtain gas was set at 0.14 MPa, atomizing air pressure was set at 0.41 MPa, and dry gas pressure was set at 0.35 MPa.

**Supplementary figures and table**

**Figure S1. Effect of contact time on the sorption amounts of CIP (a) and the pseudo-second-order kinetics model (b). Each point is the mean of three replicates.**


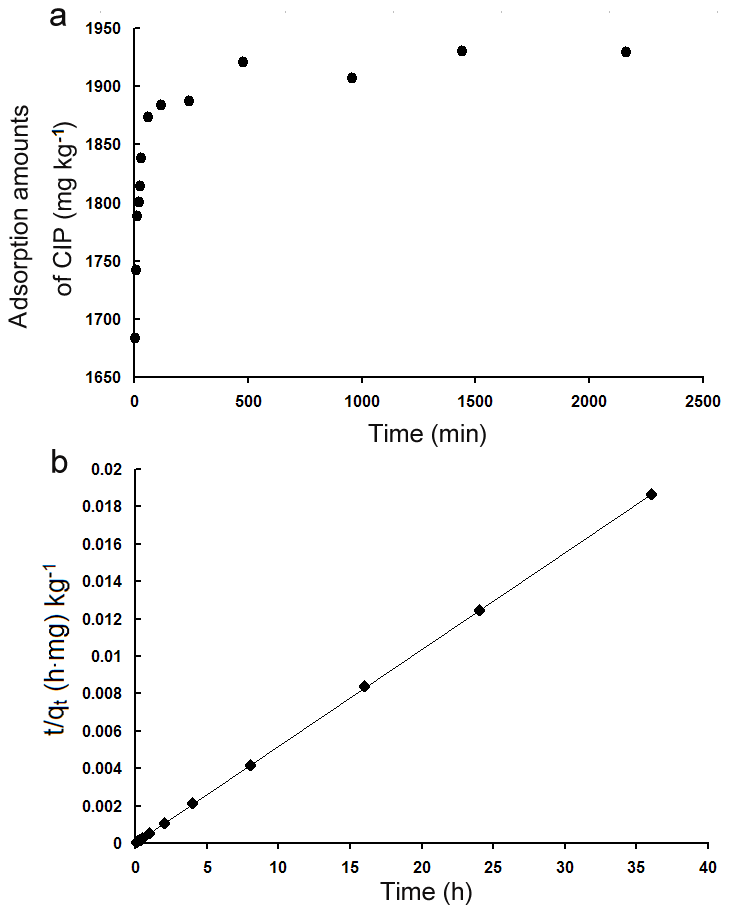


**Figure S2. Effect of CIP initial concentration on the sorption amount of CIP onto soil. Each point is the mean of three replicates.**


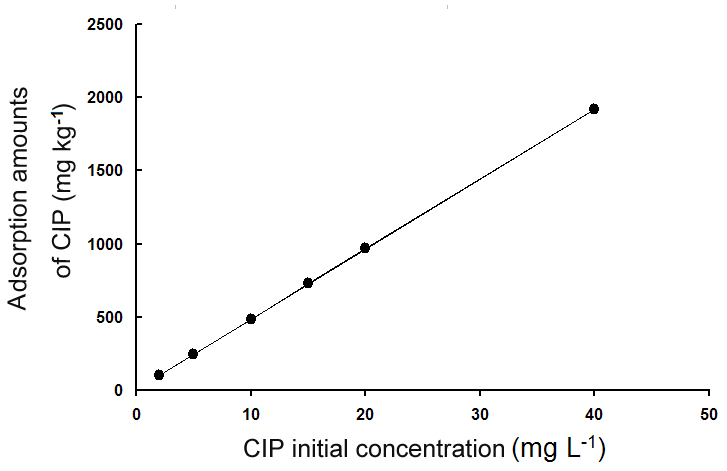


**Figure S3. The zeta potential of soil in aqueous solution as a function of solution pH.**


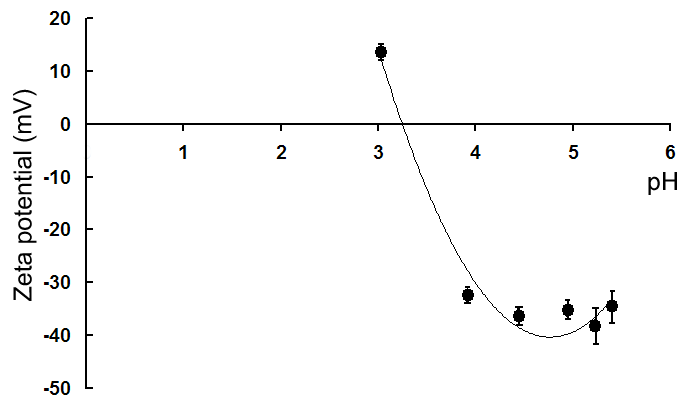


**Figure S4. Structure of the ciprofloxacin (CIP) molecule (a) and the pH-dependent speciation of CIP (b).**


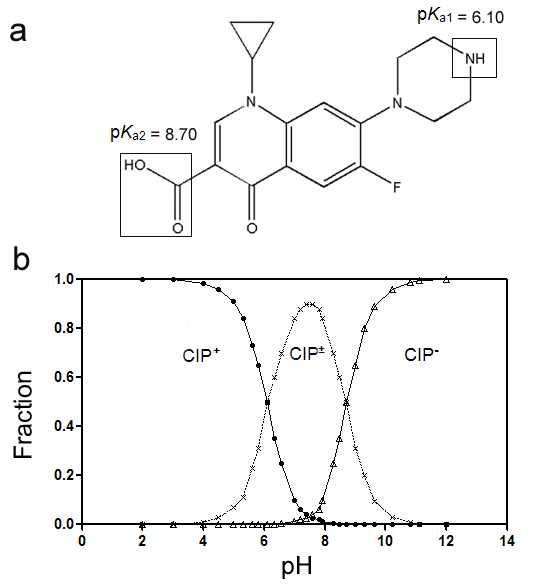


**Table S1. Effect of low-molecular-weight organic acid (LMWOA) addition on pH in the equilibrium solution with different initial loadings of ciprofloxacin (CIP) in soil.**

| CIP initial loading  (mg kg^−1^) | LMWOA addition  (g L^−1^) | pH | |
| --- | --- | --- | --- |
|  |  | Maleic acid AREs | |
| 99.1 | 0 | 6.30±0.01 | 6.30±0.01 |
|  | 0.05 | 5.38±0.02 | 5.37±0.05 |
|  | 0.1 | 5.07±0.02 | 5.01±0.01 |
|  | 0.5 | 3.20±0.01 | 3.56±0.02 |
|  | 1 | 2.46±0.01 | 2.89±0.01 |
|  | 2 | 2.07±0.02 | 2.45±0.01 |
| 245.7 | 0 | 6.31±0.07 | 6.31±0.07 |
|  | 0.05 | 5.37±0.01 | 5.33±0.10 |
|  | 0.1 | 5.04±0.00 | 5.04±0.02 |
|  | 0.5 | 3.19±0.03 | 3.58±0.03 |
|  | 1 | 2.45±0.00 | 2.88±0.01 |
|  | 2 | 2.06±0.01 | 2.46±0.01 |
| 487.4 | 0 | 6.18±0.02 | 6.18±0.02 |
|  | 0.05 | 5.37±0.02 | 5.41±0.03 |
|  | 0.1 | 5.03±0.02 | 5.08±0.01 |
|  | 0.5 | 3.17±0.07 | 3.63±0.02 |
|  | 1 | 2.50±0.03 | 2.92±0.03 |
|  | 2 | 2.07±0.01 | 2.48±0.01 |
| 729.0 | 0 | 6.19±0.03 | 6.19±0.03 |
|  | 0.05 | 5.37±0.01 | 5.40±0.02 |
|  | 0.1 | 5.06±0.03 | 5.10±0.02 |
|  | 0.5 | 3.22±0.02 | 3.62±0.05 |
|  | 1 | 2.48±0.02 | 2.91±0.04 |
|  | 2 | 2.06±0.01 | 2.48±0.01 |
| 970.9 | 0 | 6.27±0.06 | 6.27±0.06 |
|  | 0.05 | 5.33±0.01 | 5.41±0.03 |
|  | 0.1 | 5.03±0.02 | 5.07±0.03 |
|  | 0.5 | 3.20±0.04 | 3.65±0.02 |
|  | 1 | 2.46±0.01 | 2.93±0.02 |
|  | 2 | 2.06±0.01 | 2.49±0.01 |
| 1918.0 | 0 | 6.11±0.01 | 6.11±0.01 |
|  | 0.05 | 5.32±0.02 | 5.38±0.02 |
|  | 0.1 | 4.94±0.04 | 5.14±0.09 |
|  | 0.5 | 3.01±0.03 | 3.64±0.04 |
|  | 1 | 2.44±0.01 | 2.94±0.01 |
|  | 2 | 2.06±0.02 | 2.50±0.02 |

**Table S2 Equilibrium concentrations (mg L^-1^) of metal cations in solution in the presence of maleic acid.**

| CIP loading  (mg kg^-1^) | Concentrations  of acid (g L^-1^) | Cu | Zn | Cd | Fe | Mg |
| --- | --- | --- | --- | --- | --- | --- |
| 99.07 | 0 | 0.040±0.002 | 0.000±0.000 | 0.037±0.001 | 0.000±0.000 | 0.593±0.014 |
|  | 0.05 | 0.054±0.001 | 0.000±0.000 | 0.044±0.004 | 0.000±0.000 | 0.381±0.040 |
|  | 0.1 | 0.044±0.007 | 0.000±0.000 | 0.046±0.004 | 0.000±0.000 | 0.693±0.007 |
|  | 0.5 | 0.066±0.002 | 0.024±0.003 | 0.047±0.002 | 0.000±0.000 | 0.683±0.032 |
|  | 1 | 0.113±0.001 | 0.029±0.002 | 0.039±0.004 | 0.495±0.045 | 0.717±0.041 |
|  | 2 | 0.135±0.001 | 0.028±0.001 | 0.044±0.004 | 0.463±0.035 | 0.394±0.018 |
| 970.93 | 0 | 0.011±0.001 | 0.000±0.000 | 0.041±0.003 | 0.000±0.000 | 0.421±0.002 |
|  | 0.05 | 0.046±0.005 | 0.014±0.001 | 0.038±0.003 | 0.000±0.000 | 0.278±0.018 |
|  | 0.1 | 0.042±0.002 | 0.021±0.001 | 0.043±0.004 | 0.000±0.000 | 0.335±0.009 |
|  | 0.5 | 0.070±0.001 | 0.108±0.009 | 0.038±0.002 | 0.000±0.000 | 0.305±0.025 |
|  | 1 | 0.121±0.003 | 0.019±0.001 | 0.040±0.004 | 0.026±0.009 | 0.212±0.010 |
|  | 2 | 0.158±0.003 | 0.066±0.000 | 0.040±0.004 | 0.075±0.015 | 0.342±0.028 |
| 1917.99 | 0 | 0.038±0.001 | 0.000±0.000 | 0.039±0.004 | 0.000±0.000 | 0.148±0.002 |
|  | 0.05 | 0.036±0.001 | 0.000±0.000 | 0.045±0.004 | 0.000±0.000 | 0.377±0.027 |
|  | 0.1 | 0.043±0.000 | 0.000±0.000 | 0.046±0.005 | 0.000±0.000 | 0.477±0.064 |
|  | 0.5 | 0.075±0.005 | 0.035±0.004 | 0.041±0.005 | 0.000±0.000 | 0.699±0.046 |
|  | 1 | 0.124±0.003 | 0.506±0.007 | 0.045±0.002 | 0.000±0.000 | 0.350±0.031 |
|  | 2 | 0.157±0.006 | 0.153±0.008 | 0.046±0.003 | 1.124±0.096 | 0.526±0.056 |
